# A Machine Learning Model for Predicting Sarcopenia Among Middle-Aged Adults: Development and External Validation

**DOI:** 10.2196/75760

**Published:** 2025-08-27

**Authors:** Hye Jin Chong

**Affiliations:** 1 Department of Nursing Sunchon National University Suncheon-si Republic of Korea

**Keywords:** machine learning, predictive model, risk factors, sarcopenia, middle aged adults

## Abstract

**Background:**

Sarcopenia is a common muscle disorder in older adults, and its early identification and management in middle-aged populations are essential for ensuring a healthier later life. Detecting sarcopenia at an earlier stage may reduce the future burden on health care systems and enhance the quality of life in older adults. Machine learning (ML) models can evaluate large datasets, identify essential variables, and find complicated correlations between input variables. However, using ML models to detect sarcopenia remains an unsatisfied need.

**Objective:**

This study aimed to develop and externally validate an ML model to predict sarcopenia risk among middle-aged adults using a nationally representative dataset.

**Methods:**

We analyzed data from 1926 participants aged 40 to 64 years and enrolled in the 2022 Korea National Health and Nutrition Examination Survey (KNHANES). Sarcopenia was diagnosed and defined based on the 2019 Asian Working Group for Sarcopenia criteria, which incorporate both low muscle mass and reduced muscle strength. Muscle mass was assessed using bioelectrical impedance analysis with cutoffs of <7.0 kg/m² for men and <5.7 kg/m² for women. Muscle strength was measured via handgrip strength using a digital dynamometer with thresholds of <28 kg for men and <18 kg for women. Participants meeting both criteria were classified as those with sarcopenia. Four ML algorithms, random forest, support vector machine, extreme gradient boosting, and logistic regression, were used to identify risk factors of sarcopenia and predict its likelihood. The top-performing model was subsequently validated in an external cohort of 2247 middle-aged adults from the 2023 KNHANES. Model performance was assessed using the *F*_2_-score, area under the curve of a receiver operating characteristic curve, and sensitivity. All analyses were conducted using Python 3.13.2 (Python Software Foundation).

**Results:**

Among the 4 models, the logistic regression model demonstrated the strongest performance, yielding an area under the curve of 0.85, a sensitivity of 0.92, and an *F*_2_-score of 0.66. External validation using the 2023 KNHANES dataset confirmed the model’s robust performance, indicating its potential for widespread applications.

**Conclusions:**

This study developed and externally validated an ML model that accurately identified sarcopenia in middle-aged adults. Leveraging data from a comprehensive national survey, our findings underscore the significance of early detection and customized interventions in midlife to mitigate sarcopenia risk and optimize long-term health outcomes.

## Introduction

Sarcopenia is increasingly recognized as a significant muscle disease characterized by progressive and generalized loss of skeletal muscle mass and function, resulting in an elevated susceptibility to falls, fractures, infections, cardiovascular diseases, disability, and mortality [1].

In 2019, both the European Working Group on Sarcopenia in Older People [2] and the Asian Working Group for Sarcopenia (AWGS) [1] updated their definitions to emphasize the clinical importance of low muscle strength and function and reduced muscle mass.

Various causes for early-life sarcopenia have been identified, and the gap between sarcopenia progression and normal processes starts early in life [3-5]. The loss of muscle mass occurs as early as age 55 years in men and 45 years in women [6]. Midlife is also a period commonly associated with the emergence or escalation of chronic diseases and metabolic syndrome, which can further exacerbate muscle loss and functional decline. Skeletal muscle mass declines by about 6% every decade beyond middle age. Sarcopenia has a prevalence of up to 29% in middle-aged adults depending on the diagnostic criteria used [7]. Increased hospitalization expenditures due to sarcopenia are considerably greater in patients younger than 65 years than in those older than 65 years [8]. In addition, sarcopenia develops gradually and without obvious signs until a substantial decrease in muscle function occurs. Therefore, it is critical to detect risk factors for early sarcopenia in middle-aged adults. Nevertheless, the majority of risk prediction models for sarcopenia have been created to work for older adults because sarcopenia is a severe form of senescence that only affects older people [3,7]. Thus, these prediction models have restricted use in midlife populations. This gap emphasizes the importance of developing and validating new, data-driven models for early detection and intervention, promoting healthier trajectories into retirement and beyond.

Machine learning (ML) models can evaluate large datasets, identify essential variables, and find complicated correlations between input variables, making them practical tools for predicting health outcomes [9]. Clinical prediction models that use ML allow for integrating many elements to predict individual outcomes, providing deeper insights into disease risk determinants and enhancing prognosis precision [10]. Leveraging data from a population-based Korea National Health and Nutrition Examination Survey (KNHANES), this study aimed to provide a reliable, generalizable ML-based tool that can be integrated into clinical or public health settings for early detection and intervention in sarcopenia for middle-aged adults. The findings of this study could help develop approaches for improving the management and treatment of sarcopenia.

This direction is substantiated by growing evidence indicating that the risk factors for sarcopenia vary meaningfully across different age groups [4,11]. Specifically, our findings suggest that low body weight and insufficient protein intake are more influential in middle-aged adults, whereas sarcopenic obesity patterns are more frequently reported in older adults [12,13]. These distinctions highlight the need for age-specific prediction models throughout the course of life.

## Methods

### Datasets

The data for this study were obtained from the KNHANES, a nationwide dataset compiled by the Korea Ministry of Health and Welfare to investigate the nutritional and overall health status of the general public since 1998. Since 2007, cross-sectional datasets of around 10,000 people have been produced annually by stratified multilevel cluster sampling to ensure the representativeness of samples and allow the findings of research to be combined [14]. In this study, we categorized all selected variables into four domains to improve clarity and interpretability: (1) demographic variables (eg, age, gender, education level, marital status, and residence), (2) clinical variables (eg, BMI category, hypertension, diabetes mellitus, hypercholesterolemia, high-sensitivity C-reactive protein (hs-CRP), number of comorbid conditions, and subjective health perception), (3) health behavioral variables (eg, current smoking, current alcohol use, average sitting time per day, protein intake, and weekly aerobic and strength exercise participation), and (4) psychological variables (eg, perceived stress and generalized anxiety) based on the Generalized Anxiety Disorder-7 (GAD-7) score. A complete list of these variables and their distributions is provided in Table 1. Feature selection was guided by a combination of theory-driven rationale and data-driven evaluation. Initially, variables were selected based on their clinical relevance to sarcopenia as supported by existing literature. Subsequently, model-based methods, including L1-regularized logistic regression (LR) and recursive feature elimination, were used to refine the feature set based on contribution to model performance. To refine variable selection beyond theoretical rationale, we applied L1-regularized LR and recursive feature elimination as supplementary strategies. These approaches helped identify variables that consistently contributed to model performance while maintaining interpretability. Because KNHANES measurement items differ slightly from year to year, we only used datasets from years that included measurements of all relevant features (2022: test and training set; 2023: external validation set). We examined data of middle-aged adults (aged 19-64 years) from 1926 participants in 2022 and 2247 in 2023 in this study. After excluding participants who were missing variables, 1119 participants in 2022 and 1359 in 2023 were included in the final analysis (Multimedia Appendix 1).

**Table 1:** Baseline population characteristics of middle-aged adults from the 2022 Korea National Health and Nutrition Examination Surveys (N=1119).

Variables			Nonsarcopenia, n=894		Sarcopenia, n=135		*P*	
Age (years), mean (SD)			54.85 (5.70)		56.61 (5.46)		<.001	
Protein intake (g/day), mean (SD)			69.94 (32.54)		61.76 (27.12)		.002	
hs-CRP^a^ (mg/L), mean (SD)			1.30 (3.79)		1.84 (7.99)		.44	
Sitting time per day (hours), mean (SD)			8.42 (5.24)		8.29 (3.27)		.69	
**BMI category, n (%)**								<.001
	Low weight	3 (0.3)		14 (10.4)				
	Normal	277 (28.2)		110 (81.5)				
	Preobesity	247 (25.1)		9 (6.7)				
	Obesity	457 (46.4)		2 (1.5)				
**Subjective health perception^b^, n (%)**								.59
	1	33 (3.4)		7 (5.2)				
	2	279 (28.4)		33 (24.4)				
	3	498 (50.6)		66 (48.9)				
	4	160 (16.3)		27 (20)				
	5	14 (1.4)		2 (1.5)				
**Current smoking, n (%)**								.68
	No	813 (82.6)		109 (80.7)				
	Yes	171 (17.4)		26 (19.3)				
**Current alcohol use, n (%)**								.007
	No	457 (46.4)		80 (59.3)				
	Yes	527 (53.6)		55 (40.7)				
**Anxiety, n (%)**								.74
	Low	945 (96)		131 (97)				
	High	39 (4)		4 (3)				
**Perceived stress, n (%)**								.50
	Low	752 (76.4)		99 (73.3)				
	High	232 (23.6)		36 (26.7)				
**Hypercholesterolemia, n (%)**								.63
	No	623 (63.3)		82 (60.7)				
	Yes	361 (36.7)		53 (39.3)				
**Hypertriglyceridemia, n (%)**								.46
	No	824 (83.7)		117 (86.7)				
	Yes	160 (16.3)		18 (13.3)				
**Hypertension, n (%)**								<.001
	Normal	381 (38.7)		78 (57.8)				
	Caution stage	73 (7.4)		10 (7.4)				
	Prehypertension	179 (18.2)		24 (17.8)				
	Hypertension	351 (35.7)		23 (17)				
**Diabetes, n (%)**								.01
	Normal	435 (44.2)		78 (57.8)				
	Prediabetes	405 (41.2)		40 (29.6)				
	Diabetes	144 (14.6)		17 (12.6)				
**Number of comorbid conditions, n (%)**								.07
	0	403 (41)		67 (49.6)				
	1-2	465 (47.3)		59 (43.7)				
	≥3	116 (11.8)		9 (6.7)				
**Marital status, n (%)**								.80
	Married	928 (94.3)		126 (93.3)				
	Single	56 (5.7)		9 (6.7)				
**Gender, n (%)**								.001
	Male	430 (43.7)		38 (28.1)				
	Female	554 (56.3)		97 (71.9)				
**Residence, n (%)**								.55
	City	783 (79.6)		111 (82.2)				
	Rural	201 (20.4)		24 (17.8)				
**Education level, n (%)**								.29
	Under elementary school	65 (6.6)		15 (11.1)				
	Middle school	85 (8.6)		10 (7.4)				
	High school	411 (41.8)		55 (40.7)				
	College or above	423 (43)		55 (40.7)				
**Weekly aerobic exercise participation, n (%)**								.27
	No	522 (53)		79 (58.5)				
	Yes	462 (47)		56 (41.5)				
**Weekly strength exercise participation, n (%)**								.11
	No	760 (77.2)		113 (83.7)				
	Yes	224 (22.8)		22 (16.3)				

^a^hs-CRP: high-sensitivity C-reactive protein.

^b^Ranked on a 5-point scale from 1 (very good) to 5 (very bad).

### Variables

For the diagnosis of sarcopenia, AWGS 2019 maintained the original cutoffs in a sarcopenia diagnosis as a bioelectrical impedance analysis <7.0 kg/m^2^ in men and <5.7 kg/m^2^ in women [15]. Predictable variables included the following 4 categories: demographic variables, clinical variables, health behavioral variables, and psychological variables. Demographic variables included age, gender, level of education, marital status, and residence. Clinical variables included hypertension, diabetes mellitus, hypercholesterolemia, hypertriglyceridemia, anemia by diagnosis or medication, hs-CRP, number of comorbid conditions, BMI categories, and subjective health perception. For clinical variables, BMI was included as an important predictor of sarcopenia. BMI categories were classified according to the World Health Organization Asia-Pacific criteria adopted by the KNHANES as underweight (<18.5 kg/m²), normal (18.5-22.9 kg/m²), overweight (23-24.9 kg/m²), and obese (≥25 kg/m²). Subjective health perception, often measured via a single self-rated health question, is a key indicator in KNHANES and internationally. This variable was recorded as 1 (very good) to 5 (very bad). Health behavioral variables included current smoking, rate of alcohol drinking per month (under 1 cup, 0; over 1 cup, 1), sitting time per day, amount of protein intake, aerobic exercise participation, and strength exercise participation. Psychological variables included perceived stress and generalized anxiety disorder evaluated using the GAD-7 scale. Perceived stress assessed how strongly respondents feel stress in daily life from 1 (very high) to 4 (none). For reporting purposes, when analyzing the data, KNHANES typically collapses 1 and 2 together as a high-stress group and 3 and 4 as a low-stress group, deriving the variable high perceived stress (yes or no) for analysis. The GAD-7 is a self-report questionnaire with 7 items. Each item is scored on a 4-point Likert scale from 0 (not at all) to 3 (nearly every day), yielding a total possible score from 0 to 21. Researchers commonly create a binary variable for anxiety disorder (yes or no) by using a cutoff of ≥10 on the total GAD-7 scale [16].

### Model Development

We sought to predict sarcopenia by first randomly partitioning our dataset into training (75%) and test (25%) subsets using the 2022 dataset and a stratified method to preserve the original class distribution of sarcopenia. To enhance generalizability and minimize overfitting, we applied 5-fold stratified cross-validation during model training. This approach preserved class distribution in each fold and ensured robust performance evaluation across splits. Furthermore, to counteract class imbalance in the training data, we applied the synthetic minority over-sampling technique (SMOTE). Unlike simple random oversampling, which merely duplicates existing minority-class instances, SMOTE generates synthetic data points by interpolating between similar minority samples, resulting in a more generalized representation of the positive class. Following SMOTE, the number of sarcopenia-positive cases in the training set achieved a balanced 1:1 ratio between positive and negative classes. To prevent data leakage and ensure fair evaluation, SMOTE was applied exclusively to the training dataset. The test and external validation sets were left unaltered to reflect real-world class distribution during evaluation.

### Model Evaluation and External Validation

We independently evaluated the predictive performance of 4 models using area under the curve (AUC), accuracy, precision, recall, specificity, *F*_1_-score, and *F*_2_-score as the primary metrics. For external validation, the same 4 models were subsequently tested on the 2023 dataset, and their predictive performances were assessed and compared using the same evaluation criteria. This approach facilitated a robust comparison of model effectiveness across the original and external validation datasets. Then, we conducted an original variable importance analysis to evaluate each predictor’s contribution to the model’s performance because assessing the significance of input features is critical in ML models [17]. In addition, a confusion matrix–based comparison was conducted for the test set and the external validation set. This method offers a detailed view of each model’s classification performance by explicitly displaying true positives, false positives, true negatives, and false negatives. By applying it to both data partitions, we could directly assess the consistency of model predictions and identify misclassification patterns. This approach complements standard summary metrics (eg, accuracy and precision) by illuminating where errors occur, thereby enhancing the overall interpretability and validating the generalizability of the models [18].

### Data Analysis

All statistical analyses were performed with Python 3.9.13 (Python Software Foundation). All continuous variables were standardized using z-score normalization, and categorical variables were 1-hot encoded. To address extreme values, particularly in hs-CRP, which exhibited high skewness, we applied percentile-based trimming by capping values at the 99th percentile. This preprocessing strategy ensured comparability across variables, mitigated the impact of outliers, and enhanced model stability. All continuous variables were normalized via z-score standardization, and categorical variables were coded as dummy features. A *t* test was performed to compare the characteristics of the sarcopenia and nonsarcopenia groups. Variables with *P* values of <.05 were defined as statistically significant.

To achieve our research objective of developing a fast and accurate predictive model for sarcopenia diagnosis in middle-aged adults, we conducted analyses as follows. Several different ML models were selected and applied to determine the model with the best accuracy and speed. We implemented LR, a traditional statistical model, to compare its performance with the relatively recently introduced ML models. Hyperparameter tuning was performed using GridSearchCV for LR and RandomizedSearchCV for tree-based models, namely, random forest (RF) and extreme gradient boosting (XGBoost). The tuning process aimed to optimize parameters such as regularization strength, tree depth, and learning rate based on validation performance. Moreover, we used several ML algorithms, including support vector machine (SVM), RF, and XGBoost. To support reproducibility, all data preprocessing and modeling steps have been documented in Multimedia Appendix 1. A deidentified version of the analysis code will be made publicly available via GitHub upon manuscript acceptance.

### Ethical Considerations

This study was approved by the Ethics Committee of Sunchon National University (1040173-202503-HR-010-02).

## Results

### Population Characteristics

The study analyzed 1119 middle‐aged adults, comprising 984 (88%) individuals in the group without sarcopenia (nonsarcopenia group) and 135 (12%) in the group with sarcopenia (sarcopenia group). As shown in Table 1, the mean age was significantly higher in the sarcopenia group (mean 56.61, SD 5.46 years) than in the nonsarcopenia group (mean 54.85, SD 5.70 years; *P*<.001); protein levels differed significantly between the groups (sarcopenia group: mean 61.76, SD 27.12 g/day; nonsarcopenia group: mean 69.94, SD 32.54 g/day; *P*=.002). Obesity was more prevalent in the nonsarcopenia group (n=457, 46.4%) than in the sarcopenia group (n=2, 1.5%; *P*<.001). Conversely, the proportion of low weight individuals was much higher in the sarcopenia group (n=14, 10.4%) than in the nonsarcopenia group (n=3, 0.3%). Health behavioral and psychosocial factors, such as current smoking (*P*=.68) and stress (*P*=.50) did not differ significantly between the 2 groups; however, drinking status did (*P*=.007). Gender showed a notable association (*P*=.001), with individuals with sarcopenia more likely to be female (n=97, 71.9%) than male (n=38, 28.1%). Hypertension (*P<*.001) and diabetes status (*P*=.011) also differed significantly between the 2 groups. Other variables, including hs‐CRP, sitting time, anxiety, hypercholesterolemia, and hypertriglyceridemia, were not significantly different between the 2 groups. Marriage status (*P*=.80), residence (*P*=.55), aerobic exercise participation (*P*=.27), and strength exercise participation (*P*=.11) similarly showed no statistical differences between the sarcopenia and nonsarcopenia groups.

### Model Evaluation and External Validation

**Figure 1:**
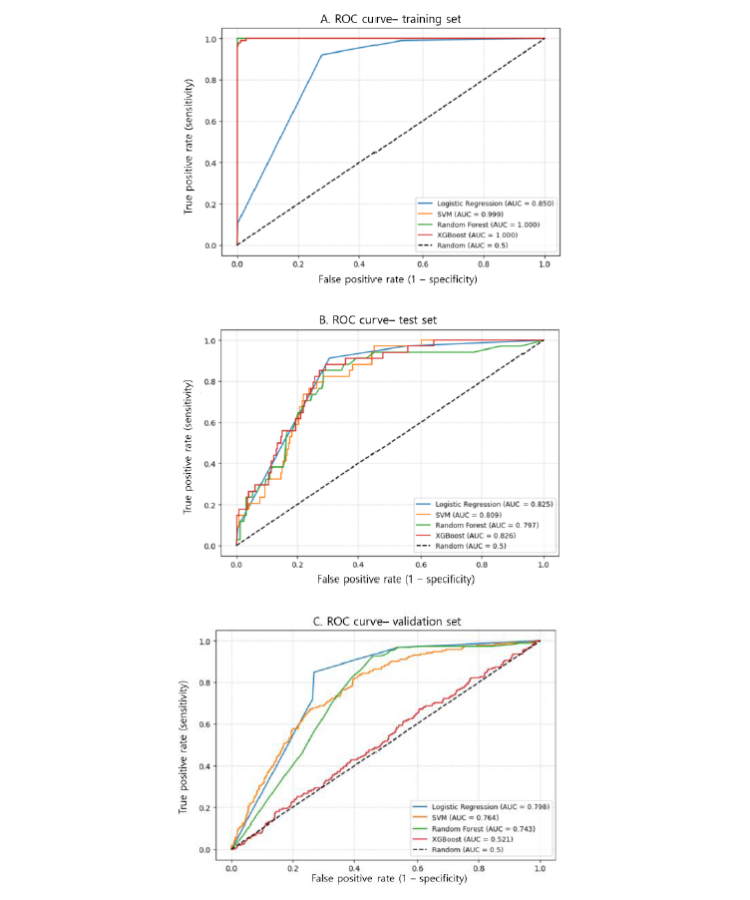
The receiver operating characteristic (ROC) curves of the predictive models. AUC: area under the curve; SVM: support vector machine; XGBoost: extreme gradient boosting.

Figure 1 and Table 2 show that in comparing 4 ML models for predicting sarcopenia, LR demonstrated the most stable performance in the test set, with an AUC of 0.82, an *F*_2_-score of 0.64, and a sensitivity of 91%. Notably, its high sensitivity and *F*_2_-score for the minority class (patients with sarcopenia) suggest a strong potential for clinical application. In contrast, SVM, RF, and XGBoost exhibited extremely high performance on the training set but significant drops in *F*_2_-score in the test set, indicating a risk of overfitting. Their sensitivities ranged from 24% to 29% in the test set, reflecting limited practical utility in detecting sarcopenia. Given its consistent performance across the training and test sets, along with advantages in identifying the minority class, LR was selected as the final predictive model in this study.

**Table 2:** Model evaluation metrics.

Metric	Logistic regression (training)	Random forest (training)	SVM^a^ (training)	XGBoos^b^ (training)	Logistic regression (evaluation)	Random forest (evaluation)	SVM (evaluation)	XGBoost(evaluation)	Logistic regression (validation)	Random forest (validation)	SVM (validation)	XGBoost(validation)
AUC^c^	0.850	1.0	0.999	1.000	0.825	0.797	0.809	0.826	0.798	0.743	0.764	0.521
Accuracy	0.746	1.0	0.995	0.993	0.721	0.864	0.839	0.825	0.748	0.141	0.840	0.141
*F*_1_-score	0.466	1.0	0.981	0.970	0.443	0.321	0.262	0.290	0.486	0.246	0.264	0.246
*F*_2_-score	0.662	1.0	0.992	0.970	0.640	0.283	0.245	0.292	0.653	0.450	0.225	0.450
Precision	0.312	1.0	0.962	0.970	0.292	0.409	0.296	0.286	0.340	0.141	0.375	0.141
Recall (sensitivity)	0.921	1.0	1.000	0.970	0.912	0.265	0.235	0.294	0.848	1.000	0.204	1.000
Specificity	0.722	1.0	0.995	0.996	0.695	0.947	0.923	0.898	0.731	0.000	0.944	0.0000

^a^SVM: support vector machine.

^b^XGBoost: extreme gradient boosting.

^c^AUC: area under the curve.

In external validation, LR exhibited stable and consistent results across the training, test, and external validation sets. Notably, it achieved an AUC of 0.80, sensitivity of 0.85, and *F*_2_-score of 0.65 in the validation dataset, indicating the most balanced capability for detecting the clinically significant minority class. In contrast, RF and XGBoost showed extremely high performance in the training set (AUC 1.00), yet demonstrated severe overfitting during validation, with a sensitivity of 1.00 but a specificity of 0.00, predicting all cases as patients. Although SVM achieved a high specificity of 0.94, its sensitivity was only 0.20 in the validation set, revealing limited utility for actual patient detection. These observations support that LR should be selected as the final predictive model in this study.

### Model Interpretation

Table 3 shows the top ten important variables. Using the LR coefficients, we evaluated each predictor’s importance by aggregating dummy-coded levels into the original variable units and calculating the mean absolute coefficient values. Obesity emerged as the most critical predictor, indicating the strongest association with sarcopenia risk in this model. Age and protein intake were also identified as influential factors.

**Table 3:** Top10 important variables based on the mean of absolute logistic regression coefficients across dummy-coded variables. This table represents the relative contribution of predictors to the model.

Variable	|Coefficient|, mean (SD)
Obesity	0.84, 0.41 (0.50)
Age	0.44, 55.06 (5.70)
Protein	0.39, 68.95 (32.04)
Drinking	0.14, 0.52 (0.50)
Hypercholesterolemia	0.14, 0.37 (0.48)
Gender	0.12, 0.42 (0.49)
Subjective health perspective	0.12, 2.85 (0.79)
Comorbidity	0.08, 0.58 (0.49)
Anxiety	0.08, 0.04 (0.19)
Hypertension	0.08, 0.33 (0.47)

**Figure 2:**
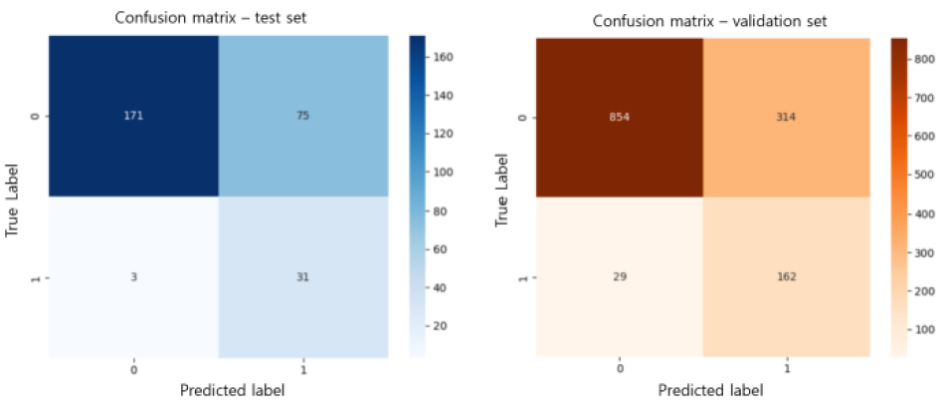
Confusion matrix for the logistic regression model.

Figure 2 presents the confusion matrices for the LR model in the test set (left panel) and external validation set (right panel). In the test set, a sensitivity (recall) of 91.2% (true positives=31, false negatives=3) and a specificity of 69.5% (true negatives=171, false positives=75) were achieved, suggesting that the model correctly identifies a high proportion of sarcopenia cases and maintains reasonable accuracy in classifying nonsarcopenia instances. A similar pattern was observed in the validation set, with a sensitivity of 84.8% (true positives=162, false negatives=29) and a specificity of 73.1% (true negatives=854, false positives=314). These results indicate that the model generalizes well, maintaining stable classification performance when applied to external data. Although SMOTE was applied only to the training set, we acknowledge that class imbalance remained in the test and validation datasets. Nonetheless, the LR model maintained high sensitivity and a stable AUC, suggesting good generalizability even under imbalanced evaluation conditions.

Table 4 provides a critical comparison of sarcopenia incidence across predicted risk categories (low, medium, high) in the test set and the external validation set, visually demonstrating the consistent classification performance and clinical applicability of the LR model. In the high-risk group, sarcopenia incidence was 29.2% in the test set and 30.8% in the validation set, reflecting a high degree of consistency and underscoring that the risk stratification threshold effectively functions in a clinically meaningful way.

**Table 4:** Observed sarcopenia incidence rate by risk group in the logistic regression model.

Predicted risk group	Test set	Validation set
Low	1.7	0.0
Medium	0.0	5.9
High	29.2	30.8

## Discussion

### Principal Findings

The primary objective of this study was to develop an ML-based model for predicting sarcopenia in middle-aged adults and to identify the optimal model among various algorithms. As a result, our study demonstrated that an ML-based risk prediction model, particularly using LR, can reliably identify sarcopenia in middle-aged adults. The LR model showed strong and consistent performance in internal testing and external validation (AUC range 0.80-0.85), underscoring its clinical applicability for early detection. One notable strength of this model is that its coefficients allow us to discern both the direction (positive or negative) and the relative magnitude of each variable’s impact, thereby providing more than just strong predictive capabilities. While interpretation tools, such as Shapley additive explanations and local interpretable model agnostic explanation, are valuable for complex models, they were not applied in this study because LR, our final model, is inherently interpretable. The model coefficients provide both direction and magnitude of each predictor’s impact, making additional posthoc interpretability tools unnecessary. This preserves clarity and enhances clinical applicability.

By elucidating how individual variables contribute to sarcopenia risk, the model offers enhanced explanatory power and actionable insights into clinical and public health interventions. Moreover, the high generalizability and strong clinical applicability of the model are evident in its consistently superior performance relative to a random classifier (AUC=0.5) across the training, test, and external validation sets. This robust and stable performance, maintained regardless of the dataset used, underscores the model’s potential for broad clinical implementation. Although direct comparisons are challenging due to a lack of research on predicting models for middle-aged adults, we were successful in developing a sarcopenia prediction model for middle-aged adults that might serve as a valuable diagnostic tool in clinical settings.

Our findings that obesity, age, and protein intake are key predictors highlight the importance of addressing modifiable risk factors before substantial muscle function decline sets in. By leveraging a large, population-based dataset, our work provides robust evidence that targeted interventions and screening in midlife can potentially mitigate the burden of sarcopenia in later life. Our findings indicate that, among middle-aged adults, lower body mass, rather than obesity, was associated with an increased risk of sarcopenia. This observation contrasts with several prior studies conducted in older populations where obesity, particularly sarcopenic obesity, has been frequently cited as a contributing factor for poor muscle function and outcomes [12,13]. However, our findings, comprising individuals in midlife, may reflect a different trajectory of muscle decline, wherein low body weight, often indicative of inadequate nutritional reserves or underlying muscle wasting, emerges as a more salient predictor [19]. Previous evidence suggests that body composition plays a distinct role depending on life stage: in older adults, excess fat mass may exacerbate functional limitations, while in younger or middle-aged adults, insufficient muscle and fat mass (as seen in underweight individuals) may indicate early sarcopenic changes [20]. Our findings thus support a life course perspective on sarcopenia, emphasizing that underweight status in midlife is not benign and may serve as a precursor to more severe muscle dysfunction in later life. This underscores the need for early lifestyle interventions aiming at maintaining healthy muscle mass and adequate protein intake before the onset of significant age-related musculoskeletal decline [21]. On the other hand, age and protein intake emerged as key predictive factors, aligning with a robust body of evidence, indicating that advancing age is a primary risk factor for sarcopenia due to age-related muscle mass decline and that insufficient protein intake significantly contributes to muscle loss and functional impairment [19,22]. Conversely, factors such as strength training, urban versus rural residence, and anemia demonstrated comparatively lower variable importance in the model, which further highlights the central role of obesity, age, and nutritional intake in sarcopenia pathogenesis and prediction [21-24].

When contextualized within prior research, our study offers several distinct advancements in the domain of sarcopenia prediction. First, while much of the existing literature has predominantly focused on older adults and has frequently relied on conventional analytical techniques, such as LR, without incorporating robust sampling strategies, our research addresses a gap by targeting middle-aged populations and using state-of-the-art ML algorithms in conjunction with SMOTE to mitigate class imbalance, an approach shown to enhance classification performance in imbalanced biomedical datasets [25,26]. Prior studies have recognized the significance of age and nutritional status in sarcopenia etiology [3], but they have often lacked external validation. In contrast, our 2-step validation strategy comprising internal cross-validation and external dataset testing strengthens the model’s generalizability and clinical utility, in line with recent calls for more rigorous validation frameworks in predictive modeling [27]. Second, conventional sarcopenia prediction tools often rely on limited indicators, such as grip strength alone. Our model adheres to the updated AWGS 2019 guidelines, integrating both handgrip strength and bioelectrical impedance analysis to provide a more comprehensive diagnostic foundation [28]. This alignment with contemporary clinical standards enhances the translational potential of our findings. Third, although muscle deterioration is known to begin in midlife, especially from the mid-40s onward [11], few studies have systematically investigated the interplay of obesity, protein intake, and age within this demographic. Our findings identify obesity and low protein intake as significant risk factors for sarcopenia in middle-aged adults, thereby extending earlier work on modifiable lifestyle components in muscle preservation [21,23]. Lastly, we demonstrate the utility of ML-based variable importance analyses, offering refined insights into the predictive strength of individual risk factors. This granularity supports the development of personalized interventions, such as tailored dietary strategies and exercise programs, for an often-overlooked age group [29].

### Limitations and Future Directions

This study has several limitations and offers opportunities for future research. First, although the large national dataset strengthens generalizability, the cross-sectional design precludes establishing causal relationships among variables. A longitudinal cohort study would be beneficial to determine temporal patterns and causation in sarcopenia onset. Second, certain lifestyle and dietary variables relied on self-reported data, which may introduce recall bias. Due to limitations in the KNHANES dataset, which lacked harmonized continuous measures of physical activity across the 2022 and 2023 waves, we adopted a categorical representation to ensure consistency and comparability across cohorts. This methodological choice is acknowledged as a limitation. Future research should integrate more objective measures, such as wearable device data, to improve accuracy. Third, while our model performed robustly overall, additional tuning or integration with newer ML techniques could further enhance prediction metrics. Larger, multicountry cohorts and diverse populations are recommended for validating and extending the applicability of our findings.

### Conclusions

Our ML-based prediction model offers a valuable framework for the early identification of sarcopenia risk in middle-aged adults, addressing a gap left by previous studies that have predominantly focused on older populations. These findings underscore the significance of proactive, personalized interventions targeting key modifiable risk factors, particularly obesity and nutrition, to mitigate muscle decline and related morbidities. By advocating for earlier detection and tailored preventive strategies, this study paves the way for improved midlife health care protocols, contributing to healthier aging trajectories globally.

## Appendix

Multimedia Appendix 1 Detailed dataset, variable definitions, and data preprocessing procedures for machine learning model development and external validation.

## Abbreviations

AUC: area under the curve
AWGS: Asian Working Group for Sarcopenia
GAD-7: Generalized Anxiety Disorder-7
hs-CRP: high-sensitivity C-reactive protein
KNHANES: Korea National Health and Nutrition Examination Survey
LR: logistic regression
ML: machine learning
RF: random forest
SMOTE: synthetic minority over-sampling technique
SVM: support vector machine
XGBoost: extreme gradient boosting

## Data Availability

All data supporting the findings of this study are available from the Korea National Health and Nutrition Examination Survey
