# Supplementary material for: A Machine Learning Model for Predicting Sarcopenia Among Middle-Aged Adults: Development and External Validation
Source: JMIR Med Inform. 2025 Aug 27;13:e75760. doi: 10.2196/75760 (PMC12423610; doi:10.2196/75760)
Supplement: Multimedia Appendix 1 [file medinform_v13i1e75760_app1.docx]

**Supplementary Appendix 1**

X = data.drop(columns=['Sarcopenia'])

y = data['Sarcopenia'] # 종속변수 (0/1)

X_train, X_test, y_train, y_test = train_test_split(X, y, test_size=0.25, random_state=42, stratify=y)

scaler = StandardScaler()

X_train = scaler.fit_transform(X_train)

X_test = scaler.transform(X_test)

from imblearn.over_sampling import SMOTE

from collections import Counter

print("Before SMOTE:", Counter(y_train))

smote = SMOTE(sampling_strategy='auto', random_state=42)

X_train_sm, y_train_sm = smote.fit_resample(X_train, y_train)

print("After SMOTE:", Counter(y_train_sm))

from sklearn.model_selection import GridSearchCV

from sklearn.linear_model import LogisticRegression

from sklearn.svm import SVC

from sklearn.ensemble import RandomForestClassifier

from xgboost import XGBClassifier

best_models = {}

# 1. Logistic Regression

log_grid = GridSearchCV(LogisticRegression(solver='liblinear'),

{'C': [0.01, 0.1, 1, 10], 'penalty': ['l1', 'l2']},

cv=5, scoring='f1', n_jobs=-1)

log_grid.fit(X_train_sm, y_train_sm)

best_models["Logistic Regression"] = log_grid.best_estimator_

# 2. SVM

svm_grid = GridSearchCV(SVC(probability=True),

{'C': [0.1, 1, 10], 'kernel': ['linear', 'rbf'], 'gamma': ['scale', 'auto']},

cv=5, scoring='f1', n_jobs=-1)

svm_grid.fit(X_train_sm, y_train_sm)

best_models["SVM"] = svm_grid.best_estimator_

# 3. Random Forest

rf_grid = GridSearchCV(RandomForestClassifier(),

{'n_estimators': [100, 200],

'max_depth': [None, 10, 20],

'min_samples_split': [2, 5]},

cv=5, scoring='f1', n_jobs=-1)

rf_grid.fit(X_train_sm, y_train_sm)

best_models["Random Forest"] = rf_grid.best_estimator_

# 4. XGBoost

xgb_grid = GridSearchCV(XGBClassifier(use_label_encoder=False, eval_metric='logloss'),

{'n_estimators': [100, 200],

'max_depth': [3, 5],

'learning_rate': [0.01, 0.1]},

cv=5, scoring='f1', n_jobs=-1)

xgb_grid.fit(X_train_sm, y_train_sm)

best_models["XGBoost"] = xgb_grid.best_estimator_

best_models

from sklearn.metrics import (accuracy_score, roc_auc_score, confusion_matrix,

precision_score, recall_score, f1_score, fbeta_score, classification_report)

results = {}

for name, model in best_models.items():

y_pred = model.predict(X_test)

y_prob = model.predict_proba(X_test)[:, 1]

auc = roc_auc_score(y_test, y_prob)

acc = accuracy_score(y_test, y_pred)

precision = precision_score(y_test, y_pred)

recall = recall_score(y_test, y_pred)

f1 = f1_score(y_test, y_pred)

f2 = fbeta_score(y_test, y_pred, beta=2)

tn, fp, fn, tp = confusion_matrix(y_test, y_pred).ravel()

sensitivity = tp / (tp + fn)

specificity = tn / (tn + fp)

results[name] = {

'AUC': auc,

'Accuracy': acc,

'Precision': precision,

'Recall(Sensitivity)': recall,

'Specificity': specificity,

'F1-score': f1,

'F2-score': f2

}

import pandas as pd

pd.DataFrame(results).T.sort_values(by="F2-score", ascending=False)

train_results = {}

for name, model in best_models.items():

y_pred = model.predict(X_train)

y_prob = model.predict_proba(X_train)[:, 1]

auc = roc_auc_score(y_train, y_prob)

acc = accuracy_score(y_train, y_pred)

precision = precision_score(y_train, y_pred)

recall = recall_score(y_train, y_pred)

f1 = f1_score(y_train, y_pred)

f2 = fbeta_score(y_train, y_pred, beta=2)

tn, fp, fn, tp = confusion_matrix(y_train, y_pred).ravel()

sensitivity = tp / (tp + fn)

specificity = tn / (tn + fp)

train_results[name] = {

'AUC': auc,

'Accuracy': acc,

'Precision': precision,

'Recall(Sensitivity)': recall,

'Specificity': specificity,

'F1-score': f1,

'F2-score': f2

}

df_train = pd.DataFrame(train_results).T.round(2)

print(df_train)

df_test = pd.DataFrame(results).T.round(2)

print(df_test)

file_path = r'C:\Users\user\P_2025_3\valid_final.csv'

valid = pd.read_csv(file_path)

y_valid = valid["Sarcopenia"]

X_valid = valid.drop(columns=["Sarcopenia"])

from sklearn.metrics import (accuracy_score, roc_auc_score, confusion_matrix,

precision_score, recall_score, f1_score, fbeta_score)

external_results = {}

for name, model in best_models.items():

y_pred = model.predict(X_valid)

y_prob = model.predict_proba(X_valid)[:, 1]

auc = roc_auc_score(y_valid, y_prob)

acc = accuracy_score(y_valid, y_pred)

precision = precision_score(y_valid, y_pred)

recall = recall_score(y_valid, y_pred)

f1 = f1_score(y_valid, y_pred)

f2 = fbeta_score(y_valid, y_pred, beta=2)

tn, fp, fn, tp = confusion_matrix(y_valid, y_pred).ravel()

sensitivity = tp / (tp + fn)

specificity = tn / (tn + fp)

external_results[name] = {

'AUC': auc,

'Accuracy': acc,

'Precision': precision,

'Recall(Sensitivity)': recall,

'Specificity': specificity,

'F1-score': f1,

'F2-score': f2

}

pd.DataFrame(external_results).T.sort_values(by="F2-score", ascending=False)

metrics = ['AUC', 'Accuracy', 'Precision', 'Recall(Sensitivity)', 'Specificity', 'F1-score', 'F2-score']

models = list(best_models.keys()) # 예: ['Logistic Regression', 'SVM', 'Random Forest', 'XGBoost']

rows = []

for metric in metrics:

for model in models:

rows.append({

'Metric': metric,

'Model': model,

'Train': round(train_results[model][metric], 2),

'Evaluation': round(results[model][metric], 2),

'Validation': round(external_results[model][metric], 2)

})

# 테이블로 변환

df = pd.DataFrame(rows)

table_all = df.pivot(index="Metric", columns="Model")

table_all.columns = [f"{col[1]} ({col[0]})" for col in table_all.columns]

table_all.reset_index(inplace=True)

table_all = table_all.round(2)

table_all

importance_df["Original_Variable"] = importance_df["Feature"].str.replace(r"_[0-9]+$", "", regex=True)

importance_df["Original_Variable"] = importance_df["Feature"].str.strip().str.replace(r"\.[0-9]+$", "", regex=True)

rename_map = {

"Subjective_Health_Perception": "SubjHealth",

"Generalized_Anxiety": "Anxiety",

"Stress_Perception": "Stress",

"Aerobic_Physical_Activity": "AerobicPA",

"Strength_Exercise": "StrengthEx",

"Hypercholesterolemia": "Hypercholesterolemia",

"Hypertriglyceridemia": "Hypertriglyceridemia",

"Sitting_time": "Sittingtime",

"Protein_Intake": "Protein",

"Obesity": "Obesity",

"Sex": "Gender"

}

importance_df["Original_Variable"] = importance_df["Original_Variable"].replace(rename_map)

grouped_importance = (

importance_df.groupby("Original_Variable")["Abs_Coefficient"]

.mean() # 또는 .sum(), .max() 등 원하는 기준으로

.sort_values(ascending=False)

.reset_index()

)

grouped_importance.columns = ["Variable", "Mean Abs Coefficient"]

print(grouped_importance.head(10))

top_vars = grouped_importance.head(10)

plt.figure(figsize=(10, 6))

plt.barh(top_vars['Variable'], top_vars['Mean Abs Coefficient'], color='steelblue')

plt.xlabel("Mean |Coefficient|", fontsize=12)

plt.title("Top 10 Important Variables", fontsize=14)

plt.gca().invert_yaxis() # 높은 값이 위에 오도록 y축 반전

plt.tight_layout()

plt.show()

model = best_models["Logistic Regression"]

y_test_pred = model.predict(X_test)

y_valid_pred = model.predict(X_valid)

cm_test = confusion_matrix(y_test, y_test_pred)

cm_valid = confusion_matrix(y_valid, y_valid_pred)

fig, axes = plt.subplots(1, 2, figsize=(12, 5))

sns.heatmap(cm_test, annot=True, fmt='d', cmap='Blues', ax=axes[0])

axes[0].set_title("Confusion Matrix - Test Set")

axes[0].set_xlabel("Predicted Label")

axes[0].set_ylabel("True Label")

sns.heatmap(cm_valid, annot=True, fmt='d', cmap='Oranges', ax=axes[1])

axes[1].set_title("Confusion Matrix - Validation Set")

axes[1].set_xlabel("Predicted Label")

axes[1].set_ylabel("True Label")

plt.tight_layout()

plt.show()

y_test_prob = best_models["Logistic Regression"].predict_proba(X_test)[:, 1]

risk_group = pd.cut(y_test_prob, bins=[0.0, 0.33, 0.66, 1.0], labels=["Low", "Medium", "High"])

df_risk = pd.DataFrame({

'Predicted_Prob': y_test_prob,

'Risk_Group': risk_group,

'Actual': y_test

})

risk_summary = df_risk.groupby('Risk_Group')['Actual'].agg(['count', 'sum'])

risk_summary['Rate'] = (risk_summary['sum'] / risk_summary['count']).round(3)

plt.figure(figsize=(7, 5))

plt.bar(risk_summary.index, risk_summary['Rate'], color=['skyblue', 'orange', 'tomato'])

plt.title('Sarcopenia Rate by Predicted Risk Group (Test Set)')

plt.ylabel('Observed Sarcopenia Rate')

plt.xlabel('Predicted Risk Group')

plt.ylim(0, 1)

plt.grid(axis='y', linestyle='--', alpha=0.6)

plt.tight_layout()

plt.show()

print(risk_summary)

y_valid_prob = best_models["Logistic Regression"].predict_proba(X_valid)[:, 1]

risk_group_valid = pd.cut(y_valid_prob, bins=[0.0, 0.33, 0.66, 1.0], labels=["Low", "Medium", "High"])

df_risk_valid = pd.DataFrame({

'Predicted_Prob': y_valid_prob,

'Risk_Group': risk_group_valid,

'Actual': y_valid

})

risk_summary_valid = df_risk_valid.groupby('Risk_Group')['Actual'].agg(['count', 'sum'])

risk_summary_valid['Rate'] = (risk_summary_valid['sum'] / risk_summary_valid['count']).round(3)

plt.figure(figsize=(7, 5))

plt.bar(risk_summary_valid.index, risk_summary_valid['Rate'], color=['skyblue', 'orange', 'tomato'])

plt.title('Sarcopenia Rate by Predicted Risk Group (Validation Set)')

plt.ylabel('Observed Sarcopenia Rate')

plt.xlabel('Predicted Risk Group')

plt.ylim(0, 1)

plt.grid(axis='y', linestyle='--', alpha=0.6)

plt.tight_layout()

plt.show()

print(risk_summary_valid)

risk_summary_test = pd.DataFrame({

'Risk_Group': ['Low', 'Medium', 'High'],

'Rate_Test': [0.017, None, 0.292]

})

risk_summary_valid = pd.DataFrame({

'Risk_Group': ['Low', 'Medium', 'High'],

'Rate_Validation': [None, 0.059, 0.308]

})

risk_comparison = pd.merge(risk_summary_test, risk_summary_valid, on='Risk_Group', how='outer')

x = risk_comparison['Risk_Group']

x_idx = range(len(x))

width = 0.35

plt.figure(figsize=(8, 5))

plt.bar([i - width/2 for i in x_idx], risk_comparison['Rate_Test'], width=width, label='Test Set', color='skyblue')

plt.bar([i + width/2 for i in x_idx], risk_comparison['Rate_Validation'], width=width, label='Validation Set', color='salmon')

plt.xticks(x_idx, x)

plt.ylim(0, 1)

plt.ylabel('Observed Sarcopenia Rate')

plt.xlabel('Predicted Risk Group')

plt.title('Sarcopenia Rate by Risk Group (Test vs Validation)')

plt.legend()

plt.grid(axis='y', linestyle='--', alpha=0.6)

plt.tight_layout()

plt.show()

dataset_names = ['Train Set', 'Test Set', 'Validation Set']

X_sets = [X_train, X_test, X_valid]

y_sets = [y_train, y_test, y_valid]

prefixes = ['A.', 'B.', 'C.']

for idx, (X, y, name) in enumerate(zip(X_sets, y_sets, dataset_names)):

plt.figure(figsize=(8, 6))

for model_name, model in best_models.items():

y_prob = model.predict_proba(X)[:, 1]

fpr, tpr, _ = roc_curve(y, y_prob)

auc = roc_auc_score(y, y_prob)

plt.plot(fpr, tpr, label=f"{model_name} (AUC = {auc:.3f})")

plt.plot([0, 1], [0, 1], 'k--', label="Random (AUC = 0.5)")

plt.title(f"{prefixes[idx]} ROC Curve - {name}")

plt.xlabel("False Positive Rate (1 - Specificity)")

plt.ylabel("True Positive Rate (Sensitivity)")

plt.legend(loc="lower right")

plt.grid(True, linestyle='--', alpha=0.6)

plt.tight_layout()

plt.show()
